# Evaluation of two semi-supervised learning methods and their combination for automatic classification of bone marrow cells

**DOI:** 10.1038/s41598-022-20651-4

**Published:** 2022-10-06

**Authors:** Iori Nakamura, Haruhi Ida, Mayu Yabuta, Wataru Kashiwa, Maho Tsukamoto, Shigeki Sato, Syuichi Ota, Naoki Kobayashi, Hiromi Masauzi, Kazunori Okada, Sanae Kaga, Keiko Miwa, Hiroshi Kanai, Nobuo Masauzi

**Affiliations:** 1 Graduate School of Health Sciences, Hokkaido University, Sapporo, Japan; 2 Graduate School of Medicine, Hokkaido University, Sapporo, Japan; 3 Department of Clinical Laboratory, Sapporo Hokuyu Hospital, Sapporo, Japan; 4 Department of Hematology, Sapporo Hokuyu Hospital, Sapporo, Japan; 5 Faculty of Health Sciences, Hokkaido University, Sapporo, Japan; 6 Graduate School of Biomedical Engineering, Tohoku University, Sendai, Japan

**Keywords:** Computational biology and bioinformatics, Image processing, Machine learning

## Abstract

Differential bone marrow (BM) cell counting is an important test for the diagnosis of various hematological diseases. However, it is difficult to accurately classify BM cells due to non-uniformity and the lack of reproducibility of differential counting. Therefore, automatic classification systems have been developed in which deep learning is used. These systems requires large and accurately labeled datasets for training. To overcome this, we used semi-supervised learning (SSL), in which learning proceeds while labeling. We used three methods: self-training (ST), active learning (AL), and a combination of these methods, and attempted to automatically classify 16 types of BM cell images. ST involves data verification, as in AL, before adding them to the training dataset (confirmed self-training: CST). After 25 rounds of CST, AL, and CST + AL, the initial number of training data increased from 425 to 40,518; 3682; and 47,843, respectively. Accuracies for the test data of 50 images for each cell type were 0.944, 0.941, and 0.976, respectively. Data added with CST or AL showed some imbalances between classes, while CST + AL exhibited fewer imbalances. We suggest that CST + AL, when combined with two SSL methods, is efficient in increasing training data for the development of automatic BM cells classification systems.

## Introduction

Bone marrow cell differential counting is a basic and important test for the diagnosis of various hematological diseases, such as myelodysplastic syndrome and leukemia^1–3^. However, it requires a lot of skill for testing and expertise to acquire the skill. Despite the difficulties involved in the test, there is non-uniformity and low reproducibility in inter and intra-observer results^3–6^. The morphological characteristics of immature or malignant blood cells differ both within a patient and among different patients. In addition, the conditions of staining may vary between specimens and facilities^7^. Furthermore, the characteristics of blood cell morphology are still defined using non-quantitative descriptions^8^.

To overcome these issues, we attempted to develop an automatic classifier for bone marrow blood cells using a deep learning system. However, a substantial amount of correctly labeled training data is required to train neural networks using deep learning (DL). Correctly labeling a large amount of data requires a significant amount of work by experts, and is time-consuming as well^9^. To overcome these difficulties, we used semi-supervised learning, which is a method for efficient labeling^10^.

Many studies have been conducted to classify bone marrow blood cells using artificial intelligence^11^. In early research, studies first analyzed and quantified morphological characteristics and then detected their differences with a discriminator such as Support vector machine (SVM)^12,13^, random binary tree (RBT)^14–17^, or other methods^18,19^. Subsequently, as the superiority of image recognition using deep learning became clear, an increasing number of studies using the technique has been reported in recent years^7,20–26^. However, DL requires preparation of a substantial amount of correctly labeled teacher data^9^, which involves a significant amount of work by experts and is time-consuming as well^9,25^. This was a major issue in system development. To solve this problem, semi-supervised learning has been developed to efficiently increase the number of teacher data using the estimation results of the model trained with a small number of teacher data^10^. Although several methods have been proposed to increae teacher data in semi-supervised learning, such as self-training (ST)^27^ or active learning (AL)^28,29^, an optimal method for classifying bone marrow blood cells has not yet been reported. Therefore, in this study we aimed at clarifying which semi-supervised learning technique is most useful in the classification of bone marrow blood cells.

## Results

**Figure 1:**
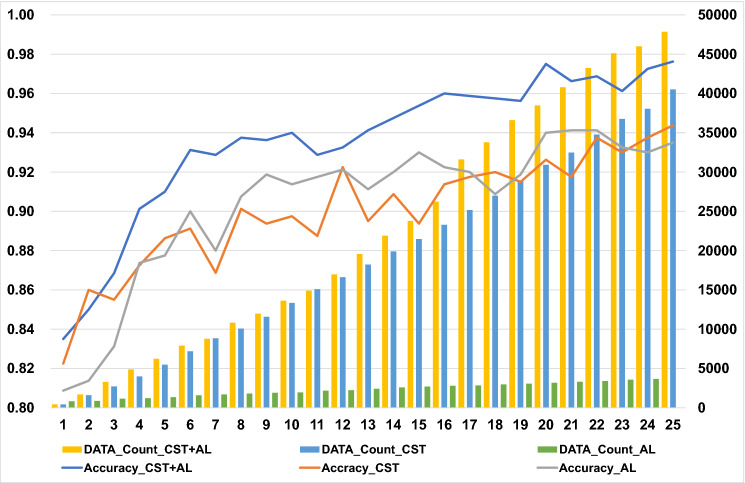
History of added data counts and accuracy for test data. In all three methods, an upward trend in accuracy was observed with an increasing number of rounds. At the 25th time of semi-supervised learning, CST had 11 times more data as compared to AL; nonetheless, the accuracy was similar. However, the accuracy was improved in CST + AL, which is a combination of the two methods. CST, confirmed self-training; AL, active learning; DATA, addition data.

The number of training data collected for confirmed self-training (CST), which is our newly improved method based on the original ST, AL, and a combination of CST and AL (CST + AL) methods, after 25 times of semi-supervised learning was 40,518, 3682, and 47,843, respectively. Table 1 and Fig. 1 show the history of an increase in the number of training data and the transition of the predicted accuracy of the test data for each learning by the semi-supervised learning method.

**Table 1:** Added data counts and accuracies after each iteration of semi-supervised learning.

Times of semi-supervised learning	CST + AL		CST		AL	
	Accuracy_CST + AL	DATA_Count_CST + AL	Accuracy_CST	DATA_Count_CST	Accuracy_AL	DATA_Count_AL
1	0.83500	425	0.82250	425	0.80875	425
2	0.85000	1694	0.86000	1603	0.81375	828
3	0.86850	3298	0.85500	2719	0.83125	876
4	0.90125	4881	0.87250	3985	0.87375	1150
5	0.91000	6236	0.88625	5487	0.87750	1227
6	0.93125	7901	0.89125	7187	0.90000	1355
7	0.92875	8783	0.86875	8852	0.88000	1597
8	0.93750	10,804	0.90125	10,082	0.90750	1701
9	0.93625	11,981	0.89375	11,572	0.91875	1814
10	0.94000	13,624	0.89750	13,332	0.91375	1909
11	0.92875	14,907	0.88750	15,079	0.91750	1963
12	0.93250	16,966	0.92250	16,619	0.92125	2172
13	0.94125	19,558	0.89500	18,227	0.91125	2250
14	0.94750	21,903	0.90875	19,889	0.92000	2420
15	0.95375	23,774	0.89375	21,470	0.93000	2593
16	0.96000	26,231	0.91375	23,284	0.92250	2711
17	0.95875	31,603	0.91750	25,163	0.92000	2791
18	0.95750	33,787	0.92000	26,997	0.90875	2843
19	0.95625	36,585	0.91500	28,886	0.91875	2974
20	0.97500	38,465	0.92625	30,930	0.94000	3073
21	0.96625	40,778	0.91750	32,493	0.94125	3178
22	0.96875	43,232	0.93750	34,766	0.94125	3304
23	0.96125	45,112	0.93000	36,761	0.93250	3395
24	0.97250	46,006	0.93750	38,051	0.93000	3574
25	0.97625	47,843	0.94375	40,518	0.93500	3682

The total number of newly labeled teacher datasets added to 17 classes of cell types after 25 rounds of semi-supervised learning (1st row of Table 2; mean ± standard error of mean: SEM; the minimum number–the maximum number) was 2383.41 ± 326.03 (384–5179; n = 17) for CST, 216.59 ± 35.06 (62–548; n = 17) for AL, and 2814.29 ± 419.22 (477–6309; n = 17) for CST + AL, respectively, among which that for AL was the smallest. The rate of increase in the total number of newly labeled teacher data by the nth round of semi-supervised learning in each class (A; 2nd row of Table 2), which was defined as the value obtained by dividing the nth number of (A) by the (n−1)th number of (A), was 1.27186 ± 0.03720 (mean ± SEM) for CST (n = 408: 17 classes × 24 rounds), 1.09893 ± 0.01268 for AL (n = 408), and 1.29402 ± 0.04291 for CST + AL (n = 408), respectively, among which there were significant differences (p < 0.0001; one-way ANOVA). The rate of increase for AL was also the smallest (Table 2). The difference of (A) between nth and (n-1)th round also significantly differed (p < 0.0001; one-way ANOVA) among the three methods, and that for AL (7.982843 ± 0.628302; n = 408) was the smallest (3rd row of Table 2). The rate of increase in teacher data was defined as the value obtained by dividing the number of increases in the data after the nth round of semi-supervised learning by 25, which was the initial number of teacher data. The average of this increasing rate by the nth round of semi-supervised learning (mean ± SEM) was 3.9307 ± 0.1599 (n = 408) for CST, 0.3193 ± 0.039 (n = 408) for AL, and 4.6488 ± 0.4281 (n = 408) for CST + AL (4th row of Table 2), respectively, among which there were significant differences (p < 0.0001; one-way ANOVA). The rate of increase in teacher data for AL was also the smallest. There were significant differences between CST and AL (p < 0.0001, Tukey–Kramer’s HSD test), and between CST + AL and AL (p < 0.0001, Tukey–Kramer’s HSD test).

**Table 2:**
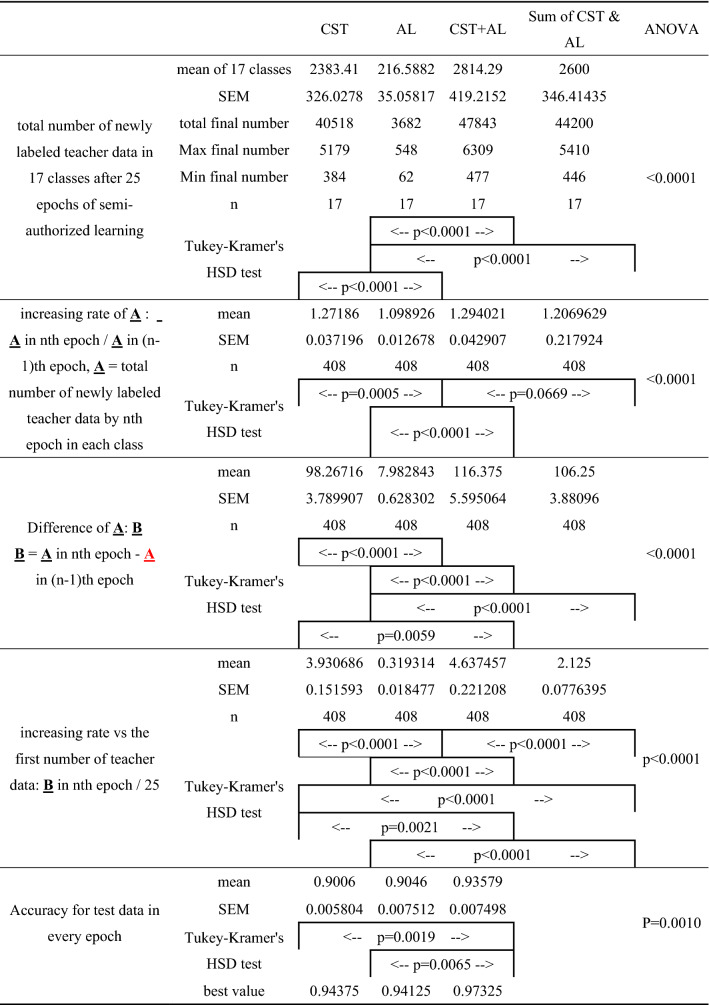
Increasing number and rate of teacher data by class and accuracy.

The total of newly labeled data by CST + AL (47,843) was more than the sum of those (40,518 + 3682 = 44,200) added only by CST and only by AL (1st row of Table 2). The average increase rate by 1 round concerning the initial number of teacher data for CST + AL (4.63746 ± 0.22121, n = 408) was larger (p < 0.0001, Tukey–Kramer’s HSD test) than the simple summation of those of CST and AL (CSL & AL; 2.125 ± 0.07764, n = 408; 4^th^ row of Table 2).

**Figure 2:**
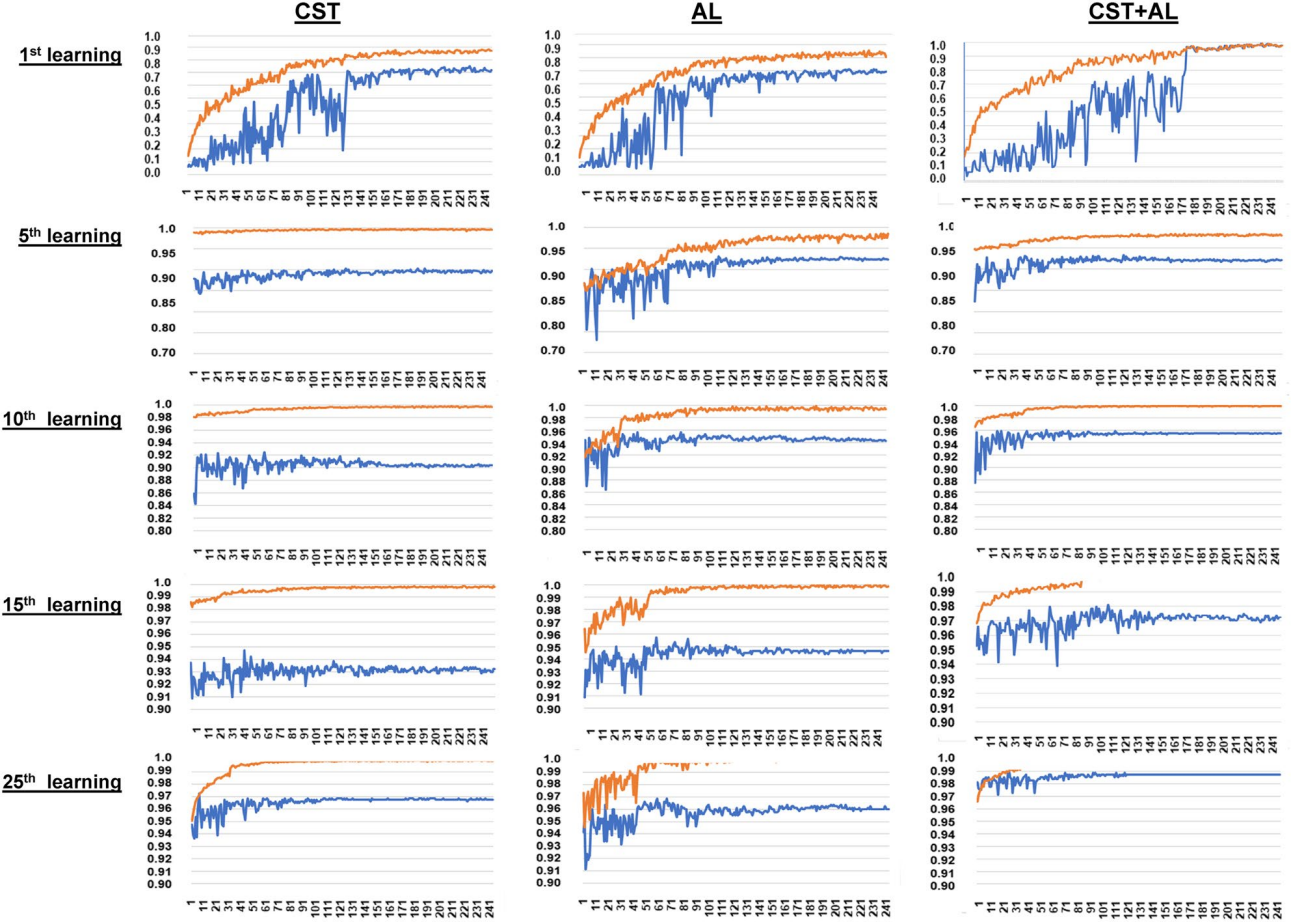
Learning curves of semi-supervised learning. The vertical axis of all learning curves indicates accuracy. The orange line indicates the accuracy for training data and the blue line indicates the accuracy for ld data. The scale of the vertical axis differs depending on the number of learnings, and the first learning was set from 0 to 1.0, 5th learning was set from 0.7 to 1.0, 10th learning was set from 0.8 to 1.0, and 15th and 25th learnings were set from 0.9 to 1.0. The horizontal axis of all learning curves indicates the count of epochs from 1 to 250.

The number of newly labeled teacher data (**C** in the 1st row of Table 3) and their differences (**D** in the 3rd row of Table 4) by 1 round among 17 classes and 25 rounds was significantly different (p < 0.0001, chi-square test) in each of the three methods (Table 3). The increase rate vs 25 (the first number of teacher data) in all classes was significantly different among the three methods, 25 rounds, and 17 classes (p < 0.0001 for all) based on multi-variable linear regression analysis (4th row of Table 3). The learning curves presented by learning and validation accuracy in the 1st, 5th, 10th, 15th, and 25th semi-supervised learning iteration with CST, AL, and CST + AL are illustrated in Fig. 2.

**Table 3:** Results of Chi-Square test and linear regression analysis for contingency tables 25 × 17 classes.

				CST	AL	CST + AL								
			N	408	408	408								
Number of newly labeled teacher data by each round in each class: **C**		Pearson's test	Chi-square	17,968.7	766.061	16,506.16								
			Prob > ChiSq	< 0.0001	< 0.0001	< 0.0001	Results of linear regression analysis using least-squares method							

			N	408	408	408		Factor	Method	epoch	Class			
			Mean	1.272223	1.104601	1.322059	Fitting of model	p (Prob >|t|)	< 0.0001	< 0.0001	0.2268	R square	F-value	p(Prob > F)
			SEM	0.037217	0.014357	0.058687						0.32286	13.7454	< 0.0001
Increasing rate of C: C in nth epoch / **C** in (n-1)th round	Linear regression analysis	vs CST + AL	EPLRLSM	0.033691	-0.1305		Linear regression analysis	LTEP	4.887	77.035	0.644			
			p (t-test)	0.1913	< 0.0001			F-value	11.07754	22.6495	1.2441			
								p (Prob > F)	< .0001	< .0001	0.2268			

			N	408	408	408								
Difference of **C** : **D D** = C in nth round—**C** in (n−1)th round		Pearson's test	Chi-square	8775.255	27,081.11	10,906.14								
			Prob > ChiSq	< 0.0001	< 0.0001	< 0.0001								

			N	408	408	408		Factor	Method	epoch	Class			
			Mean	3.930686	0.319314	4.637457	Fitting of model	p (Prob >|t|)	< 0.0001	< 0.0001	< 0.0001	R square	F-value	p(Prob > F )
			SEM	0.151593	0.018477	0.221208						0.52295	30.8507	< 0.0001
Increasing rate vs 25 (the first number of teacher data) in all the class: **D** in nth round in all classes/ 25	Linear regression analysis	vs CST + AL	EPLRLSM	0.964412	-2.64696		Linear regression analysis	LTEP	114.282	11.863	81.832			
			p (t-test)	< 0.0001	< 0.0001			F-value	331.4837	4.6573	32.6611			
								p (Prob > F)	< 0.0001	< 0.0001	< 0.0001			

Prob: Probability, ChiSq: Chi-square, SEM: Standard Error of Mean, EPLRLSM: Estimated parameter by linear regression analysis with the least square method, LTEP: Logarithmic transformed estimated parameter.

**Table 4:** Diagnosis and number of captured images for each of the 47 specimens.

Training data	No	1	2	3	4	5	6	7	8	9	10	Subtotal
	Dx	Normal	Normal	Normal	DLBCL	Normal	Normal	Normal	Normal	Normal	Normal	
	Count	19	327	837	212	148	191	438	594	229	131	3126
	No	11	12	13	14	15	16	17	18	19	20	Subtotal
	Dx	Normal	Normal	APL	Normal	MDS	CMML	CML	AML-M1	CML	MM	
	Count	177	221	102	456	328	170	280	20	734	111	2599
	No	21	22	23	24	25	26	27	28	29	30	Subtotal
	Dx	MM	MM	Anemia	PNH	CAD	MDS	MDS	MDS	MDS	MDS	
	Count	7	178	50	329	58	317	50	111	159	3	1262
	No	31	32	33	34	35	36	37	38	39	40	Subtotal
	Dx	MDS	MDS	MDS	MDS	CML	CML	CML	MDS	MA	Anemia	
	Count	361	107	189	223	369	259	163	289	80	136	2176
	No	41	42	43	Subtotal							Total
	Dx	MDS	MM	AML-M4								
	Count	141	17	20	178							9341
Test data	No	1	2	3	4	Total						
	Dx	Normal	Normal	AML	AIHA							
	Count	769	80	7	102	958						

Dx, Diagnosis; DLBCL, diffuse large B-cell lymphoma; APL, acute promyelocytic leukemia (FAB-M3); MDS, myelodysplastic syndromes; CMML, chronic myelomonocytic leukemia; CML, chronic myeloid leukemia; AML-M1, acute myeloid leukemia; FAB-M1, PNH: paroxysmal nocturnal hematuria, CAD: cold agglutinin disease, MM: multiple myeloma, AML-M4: acute myelocytic leukemia FAB-M4, MA: megaloblastic anemia, AML: acute myelocytic leukemia, AIHA: autoimmune hemolytic anemia.

The mean accuracy of CST + AL (0.93579 ± 0.007498, n = 25) for the test data (5th row of Table 2) was significantly higher (p = 0.001, One-way ANOVA, p = 0.0019 for CST; p = 0.0065 for AL, Tukey–Kramer’s HSD test) than that of CST (0.9006 ± 0.0058, n = 25) and AL (0.9046 ± 0.00751, n = 25) during 25 rounds of semi-supervised training. Yet, there was no significant difference in accuracy between CST and AL. The best value of accuracy for the test data of CST, AL, and CST + AL was 0.94375, 0.94125, and 0.97625, respectively, such that the accuracy for CST + AL was the best.

**Figure 3:**
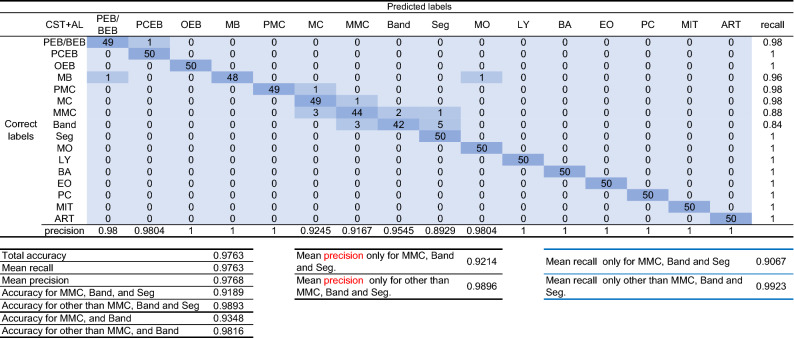
Confusion matrix for the prediction results by the model with the best accuracy. The confusion matrix was created with estimation results for the test data by the CST + AL classification model after the 25th training, which had the best accuracy. PEB/BEB, proerythroblasts/basophilic erythroblasts; PCEB, polychromatic erythroblasts; OEB, orthochromatic erythroblasts; MB, myeloblasts; PMC, promyelocytes; MC, myelocytes; MMC, metamyelocytes; Band, band neutrophils; Seg, segmented neutrophils; MO, monocytes; LY, lymphocytes; EO, eosinophils; BA, basophile/mastocyte; PC, plasma cell; MIT, mitotic-cell; ART, bare nucleus/ artifact.

The confusion matrix obtained from the CST + AL classification system after the 25th training, which had the best accuracy, is shown in Fig. 3. The average recall and average precision for the test data were 0.97625 and 0.97684, respectively. In the confusion matrix (Fig. 3), many misjudgments were observed among metamyelocyte (MMC), band-formed neutrophils (Band), and segmented neutrophils (Seg). The average recall, average precision, and accuracy for these three classes were 0.9067, 0.92136, and 0.91892, respectively, which were smaller than those for all 17 classes and those for 14 classes except for three classes (Fig. 3).

## Discussion

The number of training data added by AL, CST, and AL + CST after 25 rounds of semi-supervised learning iterations increased in the order AL + CST (47,843), CST (40,518), and AL (3682). CST added approximately 11 times as many data as AL; nonetheless, the accuracy was comparable to each other. This result suggests that increasing the teacher data does not necessarily contribute to the improvement of accuracy. It is presumed that the independence of morphological futures of teacher data is an important factor. The details of the added data for each of the three methods are shown in Supplementary Tables S1–S3.

Even in the model by CST + AL that showed the best accuracy (0.97625) in this study, the accuracy (0.93478) for MMC and Band (Fig. 3) was lower than that (0.98164) of the class excluding them. To investigate the cause of this difference in accuracy, we searched for the number of MMC and Band images added to the teacher data by each method. The mean number of metamyelocytes added by CST (62.875 ± 84.940) was smaller than the average (98.267 ± 18.700) of all classes (Supplementary Table S1). This is probably because there were few metamyelocyte images in which the estimated probability for the class output by the classifier was 0.99 or higher in the CST group. Even for human examiners, metamyelocytes are often difficult to discriminate from myelocytes and band neutrophils. In contrast, the number of added metamyelocytes (15.708 ± 11.709) and band neutrophils (15.13 ± 10.94) by AL was larger than the average (7.9828 ± 4.4619) of all classes (Supplementary Table S2). This result may be due to the characteristics of margin sampling, which more often selects images that are difficult to identify for the classifier. In other words, metamyelocytes are considered a class difficult to judge for the classifier. In CST + AL, the number of added metamyelocytes (136.88 ± 117.45) and band neutrophils (142.13 ± 106.45) was larger than the average (116.22 ± 51.914) of all classes (Supplementary Table S3). Since atypical morphological cells of both metamyelocytes and band neutrophils are also added by AL to the training data in AL + CST, it is considered that the increase of both cell types in the training data was promoted. These results suggest that the combined use of CST and AL enables efficient labeling, even for cell types that are difficult to distinguish. The mean increasing rate of metamyelocytes after each round of semi-supervised learning by CST + AL (5.475 ± 0.9584961) was 2.1769 times that of CST (2.515 ± 0.6937623). Similarly, band neutrophils were 1.452 times higher than that in CST. According to the above results, the number of MMC and Band images added to the teacher data by CST + AL was nearly twice as large as the average of classes other than these two. It is suggested that the imbalance in the number of additions for each class would not contribute to the inaccuracy. Instead, we consider the following as the cause of inaccuracies.

In this study, we used the discrimination criterion of the Blood Cell Morphology Standardization Subcommittee (BCMSS) of the Japan Society of Laboratory Hematology (JSLH)^30^ for immature granulocyte cells. The cells are defined by these criteria as follows: "the nuclei of the myelocyte are round in shape, and that of the metamyelocyte is concave (the major to short axis ratio is less than 3:1), and the nuclei with a larger or equal ratio of 3:1 or greater are band neutrophils”. The criteria define mature neutrophils as follows: "Their nucleus is segmented by chromatin filaments, of which the minimum nuclear width is less than 1/3 of the maximum width of the short axis of the nucleus or less than 1/4 of RBC diameter (about 2 μm).” Even with the model after 25 rounds of semi-supervised learning using the CST + AL method, which showed the best accuracy in this study, misjudgment was observed among the three types of granulocyte immature cells, metamyelocytes, band neutrophils, and segment band neutrophils. They are frequently found to be metamyelocytes with a laterally elongated nucleus and band neutrophils with constrictions. In the identification of these cell types, the ratios of the major axis to the minor axis of the nucleus and the ratio of the maximum width to the minimum width of the minor axis of the nucleus are important characteristic indicators. Because deep learning makes a judgment based on the morphological characteristics of the entire image, it is considered that the discrimination of these cell types based on the criteria of JSLH BCMSS was not a good definition for deep learning.

The data addition ratio in classes of eosinophils, basophils/mast cells, and plasma cells was also smaller in AL than in the other methods. Nonetheless, few misclassifications in these cell classes were observed, even with a small amount of training data. In actual values, the total number of training data points from myelocytes to band neutrophils after 25 learning sessions was approximately 300–500, and that for eosinophils, basophils, and plasma cells were 68, 70, and 77, respectively. These cells have a characteristic morphology that is easy to distinguish by human observation, and the identification rate of these cells by human observation is high as well. The high prediction probability of these cell types is an interesting result, given that deep learning has been developed with the human central nervous system as a model.

To develop a system with higher performance, we have to study and create algorithms other than deep learning that measure the diameters of the nucleus and calculate the ratios of the major axis to the minor axis of the nucleus and that of the maximum width to the minimum width of the minor axis of the nucleus. We must also work on the classification of proerythroblasts and basophilic erythroblasts, which were not distinguished due to limitations of hardware performance, and basophils and mast cells could not be separated into different classes, due to the small number of cells in blood samples.

Despite the above-mentioned limitations, we propose, based on the results of this study, that a semi-supervised learning method combining active training and confirmed self-training is a better tool for practical and rapid enlargement of training data to create an automatic blood cell recognition system than a single application of each method. We believe that the results of our study will be useful for promoting the development of practical systems in the future.

## Materials and methods

### Sample collection

Forty-three anonymized bone marrow smears, donated by Sapporo Hokuyu Hospital, were used in this study. In Sapporo Hokuyu Hospital, written informed consent for the test was obtained from all patients undergoing bone marrow aspiration examinations. Normally, in bone marrow aspiration tests, a maximum of ten smears, which exceeds the regularly required number of smears for routine examination, are prepared and preserved for additional tests such as special cytochemical staining and immunostaining that may be required later. However, some smears that remain unused after a month of storage are discarded. Such discarded unstained smears were provided to our laboratory with only a pathological diagnosis attached, and removing all patients’ identifiable data. The Institutional Review Board of Sapporo Hokuyu Hospital and the Ethics Committee of the Faculty of Health Sciences from Hokkaido University approved this study as a retrospective observational study because it included only a collection of smears and their respective diagnosis from medical records. Therefore, both the ethical review committees instructed us and the staff of Sapporo Hokuyu Hospital to display information posters of this study, and waived consent from all bone marrow aspiration patients in the present study as it was already obtained by Sapporo Hokuyu Hospital. This study was conducted in accordance with approved guidelines at both institutions. The smears were stained with May–Grunwald Giemsa (MG) staining using the standard method. Images were captured using CellaVisionDM96 in digital slide mode. Microscopic field images (901 × 823 pixels) were selected from the digital slide set. Only images that well focused several cells and with negligible overlap were selected. Each image was cropped for training data to a 704 × 704 pixels square image containing as many cells as possible. Four newly donated bone marrow smears were stained, and microscopic field images were captured using the same method as that for training data. These were used as test images. The detailed counts of the specimens and the number of square images obtained are listed in Table 4.

### Segmentation of single-cell images from microscopic field images

**Figure 4:**
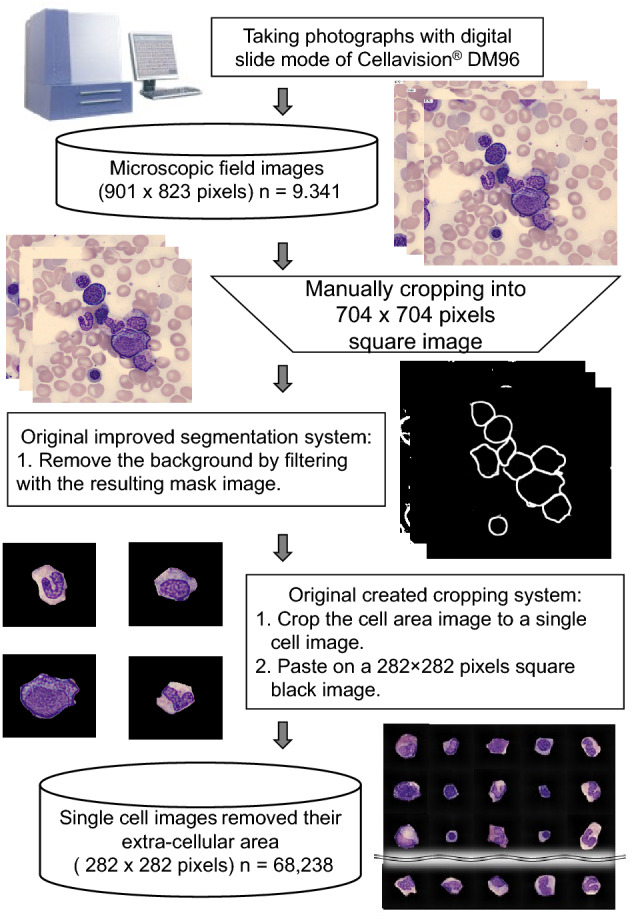
Flow of preprocessing and sample images. Microscopic field images (901 × 823) were taken with an automatic microscope (CELLAVISION DM 96). They were then cropped to 704 × 704 square images for input to the cell segmentation neural network. Square images were then input into the system to obtain 282 × 282 individual single-cell images. The inverted trapezoidal frame shows manual processing. The rectangular frame shows automatic processing or processing by hand-made programs written in Python and Cellavision DM96.

To separate the part of the image containing the cell from the background, we developed an original improved cell segmentation system based on U-Net, which is a neural network architecture for image segmentation^31^. We also created a program to crop 282 × 282 single-cell images, which automatically removed the extracellular area. The cell segmentation systems are connected to each other. This combined system crops out a single-cell region from the microscopic field image. Using this system, we segmented 68,238 cell images from 9341 microscopic field images. The segmented images included images with incomplete cell separation and some images with only cell fragments. The above process and details of the segmentation and cropping systems are illustrated in Fig. 4.

### Labeling of each cell image

**Figure 5:**
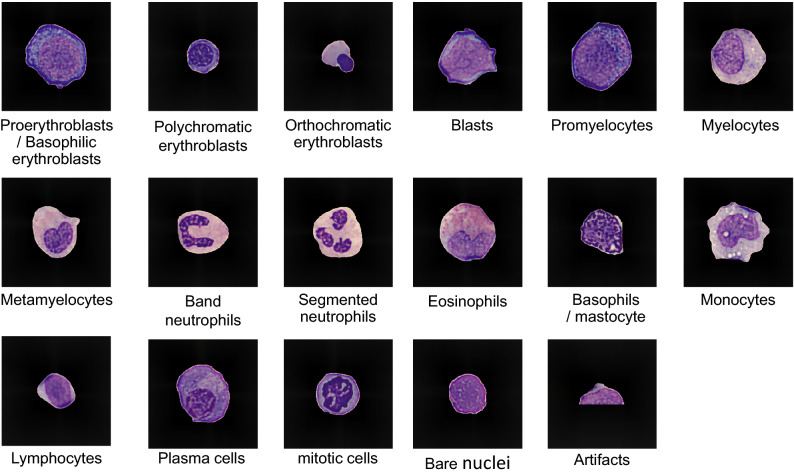
Flowchart from blood films to image data. The inverted trapezoidal frame shows manual processing. The rectangular frame shows automatic processing or processing by hand-made programs written in Python and Cellavision DM96. PEP/BEB: proerythroblast/basophilic erythroblast,

In this study, 17 types of object classes were defined for labeling: proerythroblast/basophilic erythroblasts, polychromatic erythroblasts, orthochromatic erythroblasts, blasts, promyelocytes, myelocytes, metamyelocytes, band neutrophils, segmented neutrophils, eosinophils, basophils/mastocytes, monocytes, lymphocytes, plasma cells, mitotic cells, bare nuclei, and artifacts (Fig. 5).

The classification of cell types from proerythroblasts to orthochromatic erythroblasts and from myeloblasts to segmented neutrophils was judged according to the discrimination criterion of the Blood Cell Morphology Standardization Subcommittee (BCMSS) of the Japan Society of Laboratory Hematology (JSLH)^30^.

Proerythroblasts and basophilic erythroblasts were labeled as the same class because the images of both types used in this study did not have sufficient image quality, and it is difficult to classify them stably and accurately by human visual observation.

**Figure 6:**
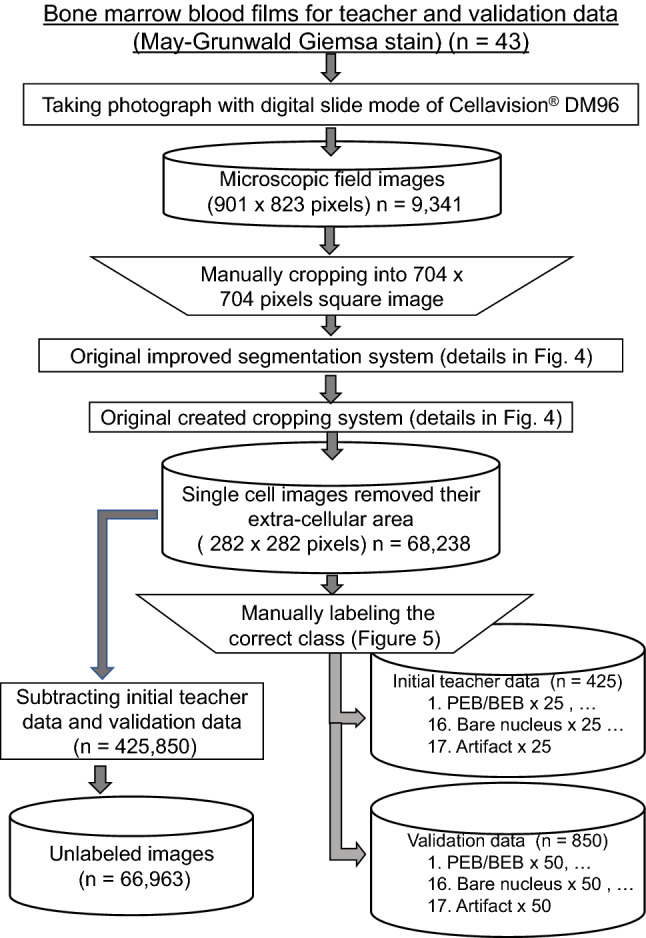
Examples of the 17 classes of bone marrow cells in this study. For classes of proerythroblast/basophilic erythroblast and basophil/mastocyte, only an image of the first label of the cell is shown in this figure.

Mast cells were labeled as the same class of basophils because only a small number were present in the microscopic field images. When we performed machine learning, the bare nuclei and artifacts were in different classes, but they were labeled as the same class in the evaluation. The above process, from bone marrow blood film preparation to creating initial teacher data, validation data, and unlabeled images, is illustrated in Fig. 6.

### Semi-supervised learning

Twenty-five images in each of the 17 classes (Fig. 5) were labeled as the initial training data. In addition, 50 images from each of the 16 classes in which bare nuclei and artifacts were combined into a class were newly labeled as validation data. First, 250 epochs of machine learning were performed using the initial training data with the architecture described in detail later. Using a deep learning model with an accuracy of 80.0% or more for the validation data after machine learning, self-training (ST)^27^ and active learning (AL)^28,29^ were performed on the unlabeled training data to obtain the new labeled training data. With these increased training data, we attempted to further improve the performance of the model.

**Figure 7:**
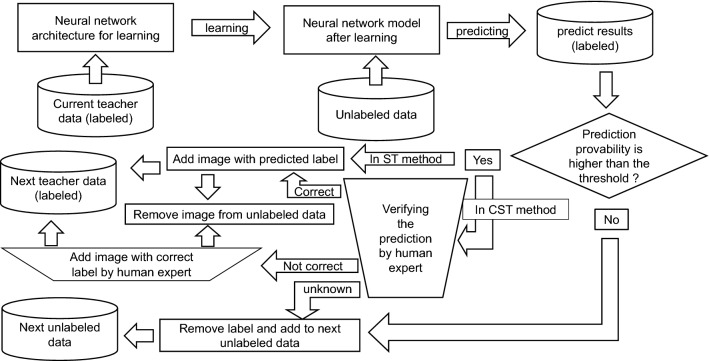
Semi-supervised learning process with self-training (ST) and confirmed self-training (CST) methods. The inverted trapezoidal frame shows manual processing or human judgment. The rectangular frame shows automatic processing by a hand-made program written in Python. The diamond frame shows the automatic judgment by a program written in python. The threshold provability in this study was 0.9.

In the ST algorithm adopted in this study, when the predicted probability of the pseudo label of certain unlabeled training data predicted by the classifier was 0.99 or more, the pseudo label was judged as the correct label on the image, and the data were added to the training data. As a result of repeating the semi-supervised learning using ST and the above rule, the accuracy was improved from 0.81625 after the first iteration to 0.84 after the third iteration. However, the accuracy subsequently exhibited a downward trend and returned to 0.81625 after ten iterations (detailed data are not shown here). ST is a robust learning method as long as every added pseudo label is correctly predicted^32,33^, but the performance of a model deteriorates when incorrectly labeled data are added to the training data^10,34^. To solve this problem, we added a new step of confirmation of the recommended data by a human observer to the ST algorithm. We named this method “Confirmed ST” (CST; Fig. 7). In this study, we evaluated the effect of increasing the number of training datasets on the improvement of performances between two types of semi-supervised methods and their combination, namely, only CST, only AL, and both of these (CST + AL). The details of CST and AL are described below.

1. CST.

In self-training, unlabeled data are provisionally labeled (pseudo-labeling) with a classifier that was first trained (machine learning) with a relatively small amount of labeled training data. These temporary pseudo-labeled data were selected according to a certain rule, in addition to the original training data. Machine learning was repeated with new training data and new training data was continuously added. As a result, the data increased step-by-step. In this study, prediction probability was used as the selection rule, and an image whose prediction probability of the pseudo label on the image was 0.99 or more was selected to be added to the training data. However, the selected pseudo-labeled data were not immediately added to the training dataset. When the pseudo label and the image were judged by human examiners and it was found that a wrong label was attached, the correct label was re-attached and added to the training data. If a candidate image was difficult to judge by the examiners, it was not added to the dataset. The above process is illustrated in Fig. 7.

2. AL.

**Figure 8:**
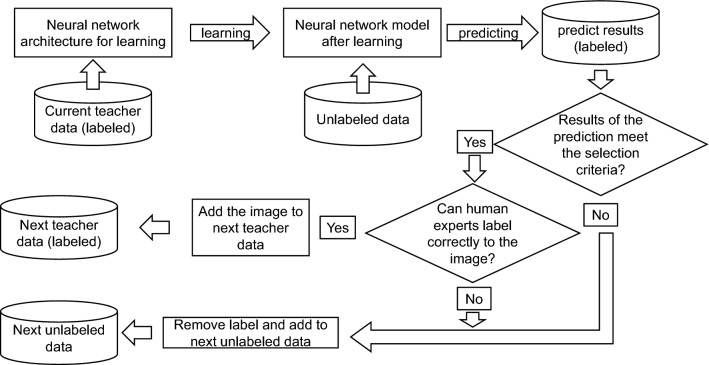
Semi-supervised learning process with the active learning (AL) method. The rectangular frame shows automatic processing by a hand-made program written in Python. The diamond frame shows the automatic judgment by a program written in python. The selection criteria of the difference in prediction provability between the highest and the second highest class was less than 0.2 in this study.

AL in machine learning is a method for efficiently increasing the number of training datasets by selecting data for addition from unlabeled data. Data are selected only if they are considered to be effective in improving the performance of a classifier. Human examiners labeled the data correctly and added them to the training data. Margin sampling was adopted as the data selection strategy. Margin sampling is a method of selecting an image in which the difference in probability between the "the most probable class" and the "the second most probable class" is less than the threshold value in the output predicted by the classifier for a certain image^29,35^. In this study, the threshold was set to 0.2. Experts correctly labeled an image for which the difference in the predicted probabilities of the top two classes was less than the threshold. The image was then added to the training data. When labeling was difficult for an image, it was not added to the training data. The above process is illustrated in Fig. 8.

3. CST + AL.

**Figure 9:**
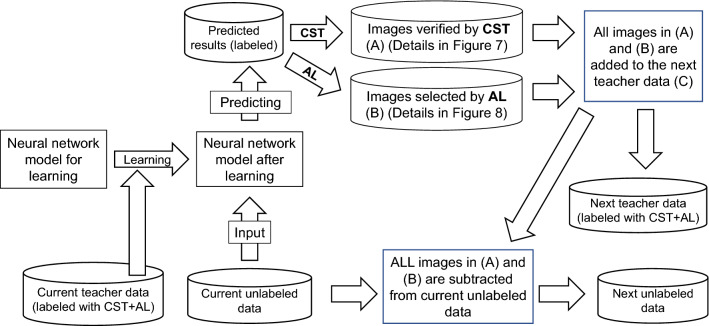
Semi-supervised learning process with the confirmed self-training and active learning (CST + AL) method. Two semi-supervised learnings, CST and AL, were performed in parallel using the same predicted labeled results. With the threshold and criteria adopted in this study, the same image was never selected as an additional candidate by both methods. All labeled images picked by both methods were added to the next set of teacher data. Then, they were subtracted from current unlabeled data to create the next set of unlabeled data. The rectangular frame shows automatic processing by a hand-made program written in Python.

We combined two methods of semi-supervised learning, CST and AL (CST + AL). Briefly, the current unlabeled data were input into a neural network model that was trained with deep learning using the current teacher data, whose number of images was increased using the CST + AL method. Candidate images for addition were filtered from the prediction result file and verified using the CST method (Fig. 9A). In parallel, the other candidate images for addition were selected by the AL method from the same prediction result file (Fig. 9B). All images in Fig. 9A, B were added to the next set of teacher data. Herein, the threshold of filtering in CST and selection criteria in AL were set to 0.9 and 0.2, respectively. Under these thresholds and criteria, it was not possible to add the same image from CST and AL to the next set of teacher data (Fig. 9C). Thus, we instead used the images (Fig. 8A, B) to create the next set of teacher data and subtracted them from current set of unlabeled data to create next set of unlabeled data. The above process is illustrated in Fig. 9.

When CST and AL are adapted to all unlabeled training data, each may select a large number of images as candidates for addition. Consequently, we randomly sampled 5000 images from all unlabeled training data in each run of the semi-supervised learning method. For each learning of the classifier, the data number for each class was adjusted every time because the learning may not be performed effectively if there is a large difference in the number of training data between classes^36^. For example, when creating the training data for the nth time, the data added in the (n−1)-th semi-supervised learning were preferentially preserved in the nth training data, and the data added before the (n−1)-th time in the labeled data pool were deleted from the nth training data to avoid exceeding 1000 images per class, if necessary. It was ensured by the above algorithm that the newly added data were used as training data at least once and that there was no large difference in the number of training data between classes during the learning iteration.

In the n-th learning, transfer learning was performed using a model with the weights of inputs for each unit of the neural network determined in the previous (n−1)-th learning. Semi-supervised learning was performed for 250 epochs at a time, and each of the three method, i.e., CST only, AL-only, and CST + AL, were repeated 25 times.

### Data augmentation

Data augmentation of the training data was applied to each machine learning operation. The input images were randomly rotated from 0° to 360°, vertically and horizontally reversed, and vertically and horizontally shifted by 12.5% of the position in every input image.

### Architecture

**Figure 10:**
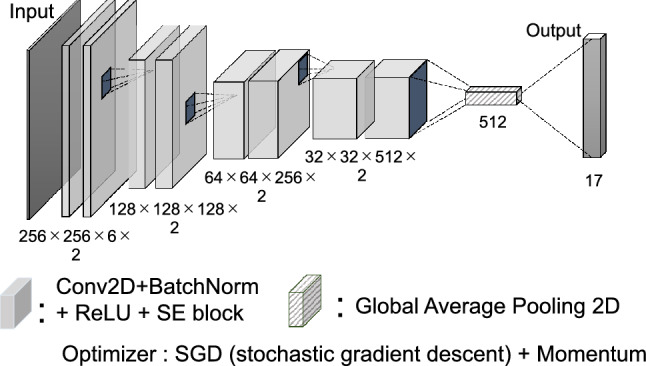
Original architecture for this study. The architecture consisted of total of eight layers of two dimensional convolution. The figures under each convolution layer indicate the size (height and width) and number of future maps and layers. “Conv2D” refers to 2-dimentional convolution. “BatchNorm” refers to batch normalization. “SE block” refers to Squeeze-and-Excitation block.

The original architecture (Fig. 10) was created and used as the classifier. This incorporated the squeeze-and-excitation block^37^ into an 8-layer architecture consisting of a convolution layer and maximum pooling. The optimization function was a stochastic gradient descent (SGD) with momentum. The learning rate was set to 0.01 for the first learning and 0.005 for subsequent learnings.

### Evaluation

To evaluate the classification system, microscopic field images from four specimens not used as training data were distributed to three qualified clinical laboratory technologists and a board-certified hematologist (Japanese Society of Hematology) in our laboratory to identify the cells in the images^31^.

We collected 50 images with the label of cell type, which was matched by four examiners from each class, except for artifacts and bare nuclei, and processed them like those used for the training data.

**Figure 11:**
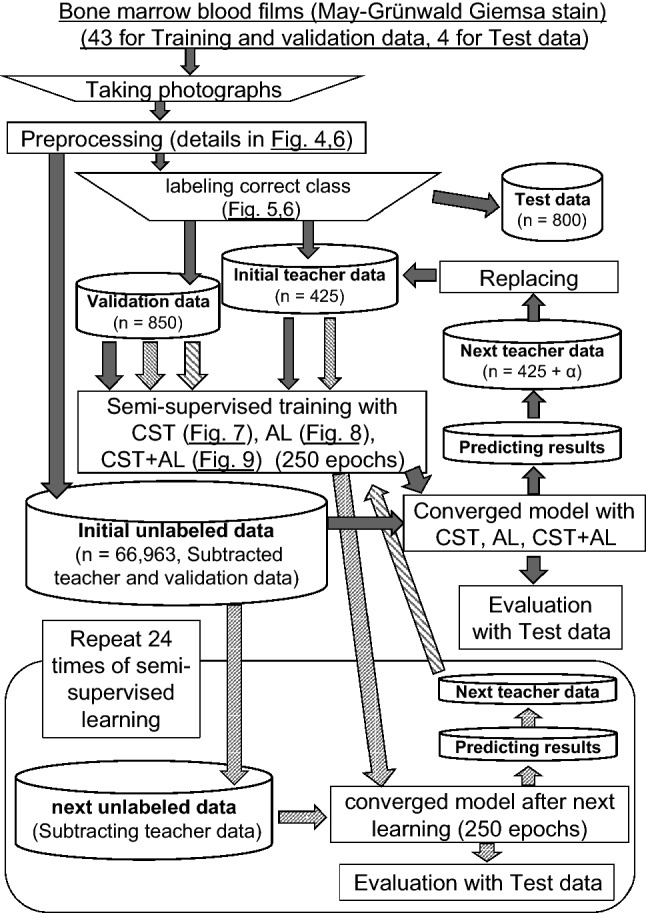
Overall flow of this study. The inverted trapezoidal frame shows manual processing or human judgment. The rectangular frame shows automatic processing by a hand-made program written in Python. The filled arrows indicate the flow in the first run of semi-authorized learning. Arrows with fine diagonal lines indicate the flow in the second run of semi-authorized learning. Arrows with rough diagonal lines indicate the flow in the third and subsequent runs of semi-authorized learning. CST: Confirmed self-training, AL: Active learning.

From images of both artifacts and bare nuclei, we collected 25 images each and created one class. The classifier was evaluated using a test consisting of 800 cell images, with the accuracy, recall, and precision. Finally, we present a flowchart of the whole procedure in this study in Fig. 11.

### Statistical analysis

The statistical analysis consisted of chi-square tests, one-way ANOVA, Dunnett’s tests, and Tukey’s multiple comparison tests using JMP® Pro 16 (SAS Institute Japan Ltd., Tokyo, Japan). Statistical significance was set at less than 5% for all two-sided *p-values*.

## Supplementary Information

Supplementary Tables.

## Data Availability

The datasets generated and/or analyzed during the current study are not publicly available due to restrictions by the Institutional Review Boards but are available from the corresponding author on reasonable request.
